# Australian consumer perspectives, attitudes and behaviours on antibiotic use and antibiotic resistance: a qualitative study with implications for public health policy and practice

**DOI:** 10.1186/s12889-017-4813-7

**Published:** 2017-10-10

**Authors:** Elaine P. M. Lum, Katie Page, Lisa Nissen, Jenny Doust, Nicholas Graves

**Affiliations:** 1 School of Public Health & Social Work, Faculty of Health, Queensland University of Technology, Kelvin Grove Campus, 60 Musk Avenue, Kelvin Grove, Brisbane, QLD 4059 Australia; 2 School of Clinical Sciences, Faculty of Health, Queensland University of Technology, Gardens Point Campus, 2 George Street, Brisbane, QLD 4000 Australia; 3 Centre for Research in Evidence Based Practice, Bond University, 14 University Drive, Robina, QLD 4226 Australia; 4 The Australian Centre for Health Services Innovation, Queensland University of Technology, 60 Musk Avenue, Kelvin Grove, Brisbane, QLD 4059 Australia

**Keywords:** Consumer, Antibiotic use, Antibiotic resistance, Qualitative, Semi-structured interview, Perspective, Attitude, Behaviour, Australia, Primary care

## Abstract

**Background:**

Consumers receive over 27 million antibiotic prescriptions annually in Australian primary healthcare. Hence, consumers are a key group to engage in the fight against antibiotic resistance. There is a paucity of research pertaining to consumers in the Australian healthcare environment. This study aimed to investigate the perspectives, attitudes and behaviours of Australian consumers on antibiotic use and antibiotic resistance, to inform national programs for reducing inappropriate antibiotic consumption.

**Method:**

Semi-structured interviews with 32 consumers recruited via convenience and snowball sampling from a university population in South East Queensland. Interview transcripts were deductively and inductively coded. Main themes were identified using iterative thematic analysis.

**Results:**

Three themes emerged from the analysis, to elucidate factors affecting antibiotic use: (a) prescription type; (b) consumer attitudes, behaviours, skills and knowledge; and (c) consumer engagement with antibiotic resistance. Consumers held mixed views regarding the use of delayed antibiotic prescriptions, and were often not made aware of the use of repeat antibiotic prescriptions. Consumers with regular general practitioners were more likely to have shared expectations regarding minimising the use of antibiotics. Even so, advice or information mediated by general practitioners was influential with all consumers; and helped to prevent inappropriate antibiotic use behaviours. Consumers were not aware of the free Return of Unwanted Medicines service offered by pharmacies and disposed of leftover antibiotics through household waste. To engage with mitigating antibiotic resistance, consumers required specific information. Previous public health campaigns raising awareness of antibiotics were largely not seen by this sample of consumers.

**Conclusions:**

Australian consumers have specific information needs regarding prescribed antibiotics to enable appropriate antibiotic use behaviours. Consumers also have expectations for high quality general practice consults conducted in a manner that increases consumer confidence in the treatment decision, regardless of whether an antibiotic is prescribed. To reduce inappropriate consumption of antibiotics and to more fully engage Australian consumers in mitigating antibiotic resistance, changes in health policy and practice are required.

## Background

Every dose of antibiotic used increases the likelihood of antibiotic resistance [1]. Antibiotic resistance occurs when bacteria change in ways that enable them to resist the effects of antibiotics, to which they previously succumbed [2]. Australia is contributing to the global problem of antibiotic resistance with an antibiotic consumption rate above the Organisation for Economic Co-operation and Development (OECD) average [3]. Successful management of antibiotic resistance requires partnerships and conscious participation of individuals including every prescriber, health professional and consumer.

In Australian primary healthcare over 27 million antibiotic prescriptions are issued annually [4]. Hence, consumers are a key group to engage in the fight against antibiotic resistance. From the literature, known barriers which prevent appropriate use of antibiotics by consumers include: confusion as to whether viruses or bacteria caused the infection [5]; the belief that antibiotics will shorten illness duration [5–9]; seeking antibiotics despite the self-limiting nature of the illness [5]; not being aware of risks associated with antibiotic use [8, 10]; needing a legitimate reason to be away from work [11]; and the perception that antibiotic resistance is a problem confined to hospitals caused by doctors who overprescribe antibiotics [12, 13].

Consumers are aware of the link between antibiotic use and antibiotic resistance [14]. However, most people misunderstood antibiotic resistance to mean that the body becomes resistant rather than the micro-organism acquiring mechanisms of resistance, to antibiotics [12, 14, 15]. Despite public health campaigns aimed at raising awareness of antibiotic resistance, a considerable proportion of consumers incorrectly think that antibiotics are effective against viral infections [16–18]. Sixty-five percent of Australian workers believe that taking antibiotics for the common cold hastens recovery and enables an earlier return to work [6]. Consumer behaviours which potentially contribute to antibiotic resistance include: seeking antibiotics for minor self-limiting illnesses e.g. acute respiratory tract infections; self-medication with antibiotics; and non-adherence to prescribed antibiotics [19, 20].

To date, qualitative studies have predominantly been conducted with consumers in Europe and the USA [7, 9, 12–15, 21–24], which have different governance, funding structures and infrastructure to that in Australia. There is a paucity of such research pertaining to the Australian healthcare environment. As a signatory of the WHO Global Action Plan [25], it is critical for Australia to have current research pertinent to its primary healthcare sector informing the implementation of its national antimicrobial resistance strategy designed to deliver an effective and sustainable response using a multi-sectoral One Health approach [26, 27].

This study aimed to investigate the perspectives, attitudes and behaviours of Australian consumers recruited from a university campus in South East Queensland in May and June (early winter) 2015 about antibiotic use and antibiotic resistance, to inform national programs for reducing inappropriate antibiotic consumption. The findings of this study are pertinent to Objective 1 of Australia’s national antimicrobial resistance strategy — to “increase awareness and understanding of antimicrobial resistance, its implications, and actions to combat it through effective communication, education and training” [27].

## Methods

### Semi-structured interviews

Pragmatism, understood as a problem-driven approach, was the underpinning philosophy for this study [28]. The one-on-one semi-structured interview was the method selected to capture the lived meanings and views of consumers, as it allowed: exploration of a main set of questions while enabling flexibility for follow-up questions; flexibility of question sequence according to how conversations unfolded; participant freedom to express views without fear of being judged by fellow participants; and minimised the likelihood that only what they deemed socially acceptable or desirable was disclosed. An interview guide for the semi-structured interviews was developed using upper respiratory tract infections as a point of conversation, as consumer misconceptions around antibiotic use for such illnesses remain [6, 12, 14–20]. Pilot interviews conducted with three consumers to refine the interview guide were not included in the data analysis. The main questions in the interview guide are shown in Table 1.

**Table 1:** Main questions in the interview guide

Sub-topics	Indicative phrasing of main questions
Self-care strategies in managing an upper respiratory tract infection	When you get a common cold or cough, what would you normally do to manage it?
Triggers for and expectations of the GP consultation	What would lead you to decide to see a GP?
	If you did go to the GP, what would be your expectations for that visit?
Views on repeat and delayed antibiotics	What is your view on repeat prescriptions? [an explanation of repeat antibiotic prescription was given]
	What is your view on delayed prescriptions? [an explanation of delayed antibiotic prescription was given]
	What do you normally do with these prescriptions?
Views on the use of antibiotics	What information or advice did the GP give you about the antibiotics?
	What information or advice did the pharmacist give you about the antibiotics?
	How much does the risk of side effects of antibiotics worry you? [examples of side effects provided if required]
	Many people find it hard to remember to take antibiotics as prescribed. What is your own experience of this?
	What do you do with leftover antibiotics?
Views on antibiotic resistance	When you hear the phrase “antibiotic resistance”, what comes to mind?
	Can you tell me in your own words what antibiotic resistance is?
	In your view, is antibiotic resistance an issue that would affect the community at large?
	What do you think can be done to manage antibiotic resistance?
	Can you think of things that you can do as a private individual that can help reduce antibiotic resistance?

#### Sampling and recruitment

Eligible participants were consumers between 18 and 54 years of age, residing or working within a 1 h drive from the Brisbane Central Business District. This age range was specified as it represents the peak years of the Australian workforce [29]; and 65% of Australian workers believe antibiotics would hasten recovery from the common cold enabling an earlier return to work [6]. Convenience and snowball sampling were used in the recruitment of participants via an email sent to university staff and students, as well as via Twitter®. Participants were recruited until no new relevant information was obtained.

#### Interview procedure

Individual interviews were conducted face-to-face and audio-recorded in May and June 2015, either at participants’ workplace or in a university meeting room. The length of interviews was between 30 and 59 min (average, 43 min). Ethics approval was granted by the Queensland University of Technology. Informed written consent from participants was obtained prior to the interview. Participants were not paid for their time, but were offered appropriate incentives in line with university guidelines. Participants were also offered a summary report of research interviews.

Interviews were conversational following Rubin and Rubin’s responsive interviewing technique [30]. Paraphrasing was used throughout the interview to clarify and confirm accurate interpretation of the intended meaning. Researcher self-reflections (EL) were documented immediately after each interview using an adapted template [31]. The interview process and content, observations of non-verbal communication, and any new concepts/themes that could be explored with subsequent participants were noted.

The quality of the co-created knowledge in an interview is contingent on the skill of the researcher (EL); where co-creation invokes the deliberate practice of the researcher in leading and being led by the participant as a conversation partner. Previous experience as a clinical pharmacist, skills in educational visiting, and active listening were used in the preparation and conduct of these interviews. The co-creation of knowledge via interviews minimises researcher bias as the backgrounds and worldviews of both researcher and participants, shape the research.

#### Data analysis

Interviews were transcribed verbatim from the audio recording, using an adaptation of the Jeffersonian Transcription Notation [32]. De-identified transcripts were uploaded to NVivo (Version 11.3.1.777) for coding and analysis [33].

Deductive and inductive coding were used to identify categories and themes. A codebook was developed *a priori* for deductive coding based on the main interview questions. An eclectic combination of coding methods was used for inductive coding: descriptive, initial, In Vivo, and theming the data [34]. To test the reliability of codes, three transcripts (1%) were randomly selected using the Microsoft Excel® random number function, for coding confirmation by another researcher (KP). The level of agreement between both researchers was high; some inductive codes were refined collaboratively.

## Results

**Fig. 1:**
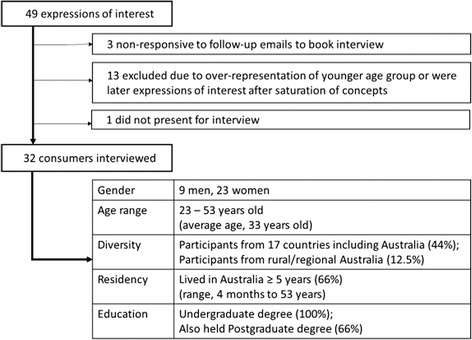
Recruitment yield, attrition, and participant characteristics

Recruitment yielded 49 expressions of interest; 32 consumers were interviewed. Fig. 1 shows recruitment and participant characteristics.

Three themes comprising nine sub-themes elucidate factors linked to the perspectives, attitudes and behaviours of Australian consumers, affecting antibiotic use and antibiotic resistance (Table 2). Each theme and its corresponding sub-themes are presented in this section.

**Table 2:** Themes and sub-themes

Themes	Sub-themes
1. Prescription type	Delayed antibiotic prescriptions
	Repeat antibiotic prescriptions
2. Consumer attitudes, behaviours, skills and knowledge	Patient expectations
	Antibiotic use behaviours
	Self-care strategies for respiratory tract infections
	Antibiotic risks
3. Consumer engagement with antibiotic resistance	Information needs and consumer education
	Views on antibiotic resistance
	Mitigating antibiotic resistance

### Theme 1: Prescription type

This theme covers consumer perspectives and the resulting influence on antibiotic use behaviours linked to the use of two types of antibiotic prescriptions — delayed and repeat antibiotic prescriptions.

#### Delayed antibiotic prescriptions

A delayed antibiotic prescription is an antibiotic prescription given by a general practitioner (GP) to a patient with instructions to use it only if symptoms worsen or do not improve in a few days. Consumers were divided on the issue of delayed antibiotics and whether this was a preferable course of action for GPs to take in instances where the need for antibiotics is uncertain. Many consumers had been given a delayed antibiotic prescription under such circumstances. Those in favour of a delayed antibiotic prescription cited personal convenience as the over-riding factor. In that, they did not have to return for reassessment should their symptoms worsen, especially in proximity to an important event such as university exams, a wedding, or travel.

Consumers not in favour of a delayed antibiotic prescription were overwhelmingly uncomfortable that the final decision whether to use antibiotics fell on them, rather than the GP. Although highly educated, these consumers did not want such responsibility. They were concerned that GPs did not, or were not able to, provide precise and definitive instructions on when to use the delayed antibiotic prescription:

“… sometimes they’re [GP] just like, ‘Oh just keep it in case you need it [delayed prescription].’ I’m like, what- ((makes an incredulous face)) … Well, how will I know if I need it?” (CS06, female, 38 years old)

Some consumers interpret the provision of a delayed prescription to mean that the GP was leaning towards the judgment that antibiotics were warranted. In such cases, consumers reported that they filled the prescription immediately (thus negating the GP’s intent for the delayed prescription) and commenced treatment without delay; unless the GP had given explicit instructions not to do so.

#### Repeat antibiotic prescriptions

A repeat antibiotic prescription enables a patient to obtain another course of antibiotics without having to consult a GP. Repeat antibiotic prescriptions are authorised by the GP when issuing the original antibiotic prescription. These prescriptions are usually only issued if: (a) the GP intentionally prescribes a duration of treatment which requires more than one course of antibiotics, (b) when the dosage required means a repeat prescription is needed to obtain the correct quantity of tablets/capsules for a course of treatment, or (c) when a standby antibiotic prescription is needed in the event of an acute relapse/exacerbation e.g. patients suffering from chronic obstructive pulmonary disease.

When GPs do not explicitly discuss what to do with repeat antibiotic prescriptions — assuming one was given intentionally, let alone those issued unintentionally due to default settings in computer-generated prescriptions — consumers are left to decide for themselves how to act. Undesirable consequences may ensue, such as truncated treatment or an unintended gap in treatment.

“I finish[ed] one [prescription] and I thought that’s all over you know, but I see that I’ve got a repeat. And so, you know, ‘Oh wait, do I need to do this?’ [fill the repeat prescription and take the antibiotics]. And then you call up [the clinic] and then by the time you get to the pharmacy again, sometimes you’ve lost time. To me, that was kind of annoying, when that happened. … I ended up getting the repeat. But it wasn’t until about three days [later], so there was a 3-day gap.” (CS02, female, 27 years old).

Unused repeat prescriptions create a problematic “reservoir” of antibiotics accessible to consumers, much like unused delayed antibiotic prescriptions. Many consumers retained these prescriptions for future use, discarding them only when the prescription can no longer be dispensed. Antibiotic prescriptions are currently valid for dispensing within 12 months from the date of prescribing.

### Theme 2: Consumer attitudes, behaviours, skills, and knowledge

This theme groups together key sub-themes related to consumer attitudes, behaviours, skills and knowledge which influence antibiotic consumption: expectations for antibiotics, how consumers use antibiotics, self-management strategies for respiratory tract infections, and consumer perception of risks associated with antibiotic use.

#### Patient expectations

Consumers who have regular GPs tend to have shared expectations regarding overall management of their health and health outcomes. Informed consumers with established doctor-patient relationships reported having had preliminary conversations regarding their preference to avoid antibiotic use where possible. Although it was not apparent whether these conversations were GP or consumer initiated, it provided a basis for future GP consultations whenever the need for antibiotic use came into question:

“But if you’re going to miss more than 1 day [of work] then I’ll go and see a doctor to get a medical certificate. … I’ll say to them to make clear that I don’t want antibiotics or anything. I don’t need to say that if I see my GP. Because she knows that.” (CS06, female, 38 years old)

Overwhelmingly, when describing what they expected from a GP consultation, consumers said they wanted to be listened to. Consumers also expected the GP to conduct a thorough clinical examination, explain their findings, treatment options, and decisions, and to answer any questions. These elements of the consultation taken together, increased consumer confidence in the professionalism, competence and trustworthiness of the GP. Most consumers would accept the GP’s decision not to prescribe an antibiotic if it was clearly explained:

“I’d be disappointed that it couldn’t be treated [with antibiotics]. But I also understand that there’s no point in treating some things with antibiotics. … So if that was clearly explained, I think I’d be less disappointed in the care that I receive from the doctor. But of course, I would always be disappointed in the fact that you just have to like, tough it out through a sickness, because there’s no easy fix.” (CS05, female, 29 years old)

#### Antibiotic use behaviours

Consumers struggled to adhere to prescribed antibiotics. The most common behaviour was not completing the course of antibiotics, either due to a conscious decision to cease taking, or forgetting to take when they felt better as the main driver of adherence (feeling ill) is lost. Given that most consumers rely on GPs’ instructions to guide them on appropriate use of antibiotics, GPs who omit to provide clear advice may inadvertently contribute to misuse. For example, omission to provide advice on the duration of treatment led to non-completion of the prescribed course of antibiotics for this consumer:

“… [the] doctor doesn’t comment much about when you stop it ((taps table twice with palm of hand for emphasis)). So, the assumption is, you stop when you feel good.” (CS16, male, 26 years old)

Middle-of-the-day doses were especially challenging to remember for antibiotics requiring a dosing frequency of three or more times a day. Some handled missed doses by working it in within the same 24-h period, doubling the antibiotic dose for their next dose, or skipping the missed dose resulting in taking longer to complete the course:

“… I have no idea what effect that has, but I figured- I guess it’s better to finish it.” (CS06, female, 38 years old)

Others were proactive in anticipating the likelihood of forgetting doses and implemented strategies, such as phone apps as reminder systems.

Consumers reported the following undesirable behaviours: using leftover antibiotics from the last unfinished course the next time they were unwell with similar symptoms, sharing unused medications with other people, and disposing of expired unused/leftover antibiotics as part of household waste:

"Just keep [the leftovers]. Like next time somebody gets sick you start with that, you don’t even go to the doctor ((chuckles))." (CS16, male, 26 years old)

" … like when I was taking them [antibiotics] all the time, I would keep them [leftovers] because I would expect to take them very soon. … and then they run out of date … and I just had a big clean out last year ((chuckles)) and I just chucked them all out. And I probably should have taken them to the pharmacy but I didn’t, I just chucked it." (CS04, female, 31 years old)

Antibiotic mixtures/syrups were disposed of in household sinks. Most consumers were not aware of the Return of Unwanted Medicines (RUM) program for safe destruction of medicines offered at no charge by community pharmacies.

Social influences were important in shaping consumer approach and behaviours in antibiotic use. Influences from family — parents (while growing up), friends and partner/spouse, were cited.

"He [husband] would just say- ‘coz I guess he’s very against all the antibiotics and everything, and I think that’s probably another influence on me that I have shifted from that, because … I would come back from [name of country, visiting family overseas] with a big sack of medication ((smiles)). And he said, 'Oh you shouldn’t be doing this you know, you have to go and see the doctor' … and he said, 'you know the more you take [antibiotics], it’s not going to work anymore…' " (CS04, female, 31 years old)

The GP was another important influence, particularly for consumers who had established good doctor-patient relationship with a regular GP. For example, consumers reported that they were more likely to complete the course of antibiotics if the GP had explicitly instructed them to do so. Another consumer recounted that patient education from her GP, enabled her to recognise and resist inappropriate behaviours i.e. not accepting shared antibiotics from family and not using her own leftover antibiotics.

The relatively higher cost of products for symptomatic management of coughs and the common cold may motivate some consumers to seek antibiotic treatment, which costs less. This was true of consumers for whom costs of healthcare was a key concern. For example, these consumers would prefer to use bulk-billing clinics as far as possible to reduce out-of-pocket costs.

“Because often I guess, those types of remedies like nasal sprays and … you know all those other types, they can … be a lot of them and they can be expensive [to purchase]. So whereas, antibiotics can just be quite affordable, in terms of treatment options … Like even just like cold and flu tablets are more expensive than antibiotics themselves.” (CS05, female, 25 years old)

#### Self-care strategies for respiratory tract infections

Consumers used the following strategies for symptomatic management of respiratory tract infections: home remedies, commercially available natural remedies, immune boosters, over-the-counter cough and cold products, increased rest and increased fluids. Consumers with underlying respiratory conditions such as asthma were especially mindful of following their asthma management plan during periods of intercurrent illness.

Workplace culture can shape self-care behaviours. Some workplaces expect staff to “power through” minor illnesses such as coughs and the common cold, whereas other organisations deem it acceptable for staff to take a few days off to recuperate at home, and to minimise the spread of infections to colleagues.

Consumers were realistic about feeling miserable and unwell during a common cold or cough, and would tolerate their symptoms for up to 3 weeks before seeking a GP consult. Self-care and self-management strategies were used in the meantime to alleviate their symptoms.

Consumers were more likely to seek a GP consult if: they were unable to take time off work/study to recover, they had to maintain a high level of functionality (both work and family commitments), they have an important upcoming event, they need a medical certificate to take time off work to recover, and persistence or worsening of symptoms.:

“… so it would depend on my symptoms, but also what I have to do [life context]. So if I’m going through a period where I don’t have a lot on, then I wouldn’t mind so much suffering through symptoms. But if I have something that I need to do like a run [athletic event] or something important job-wise or something [like that], then I might seek out healthcare earlier. … I’ll just say persistence of the symptoms.” (CS05, female, 29 years old)

#### Antibiotic risks

Many consumers perceived antibiotics to be safe medicines; and hence, would lean toward taking the antibiotic if a delayed prescription was given. Others expressed their preference to avoid antibiotics where possible due to being unsure how the antibiotics worked, unwanted effects it could have on the body, and being concerned about becoming “resistant”. Many of these consumers considered themselves low users of medicines overall.

“I’m pretty hesitant to take them [antibiotics], only because of the lack of information that I have received about what they actually do. And I prefer to find an alternative method to fixing what ails me ((chuckles)).” (CS02, female, 27 years old)

Cultivating a healthy immune system was another reason cited by consumers who were mindful of using antibiotics only when required:

“… from a while ago I got the idea in my head that in order to build up your immune system well, you need to give it a chance to fight things on its own. It’s only if it’s clearly going to lose the battle that you should really help it with medication.” (CS10, female, 23 years old)

Consumers who had negative experiences with antibiotics were more reluctant to use them unless necessary. They reported experiencing side effects such as: adverse impact to digestive system for a prolonged period, feeling physically tired, and vaginal thrush. Consumers who had neutral experiences with taking antibiotics, for example those with no troublesome side effects and recovered with the treatment, were open to using antibiotics.

### Theme 3: Consumer engagement with antibiotic resistance

This theme comprises three sub-themes which has an impact on consumer engagement with the fight against antibiotic resistance.

#### Information needs and consumer education

Consumers interviewed recognised that individuals can contribute to the fight against antibiotic resistance through responsible use of antibiotics. To help them do this, consumers wanted the following types of information: how the antibiotic worked; the rationale for selection of an antibiotic (quote 1); the duration of treatment, including when or whether the repeat antibiotic prescriptions (if issued) should be used; whether they can have alcohol while on antibiotics (quote 2); and the rationale for finishing the course of antibiotics or the consequences of not doing so, to encourage adherence to the treatment. Consumers noted that their information needs were often not voluntarily met by GPs or community pharmacists.

Quote 1: “But it seems to be the same one that’s prescribed. So it would just be good if they [the GP] could provide the information of how that actually helps an ear infection and chest infection. You know … because they’re completely different. To me they’re completely different areas [the infections].” (CS09, female, 31 years old)

Quote 2: “And so I looked it up because I have heard competing information. … And all of the information that I could find said, most antibiotics, doesn’t really do anything when you drink alcohol [not necessary to avoid alcohol], but these particular like, maybe two strains or something … stop you from processing alcohol properly … so … you only need to have one drink or less, and you can start vomiting and fainting and be really sick ((chuckles)). So the doctor didn’t tell me that much detail. He just said, don’t drink alcohol.” (CS18, female, 29 years old)

Consumers interviewed were largely not aware of the public campaigns, whether current or past, run by Government or Commonwealth funded agencies. Some were generally sceptical about news reported in mainstream media, and assumed that the issue of antibiotic resistance had been sensationalised. However, consumers were consistent in acknowledging that antibiotic resistance is a difficult topic with which to engage the public, as it is “invisible” and inconsequential for most people currently:

“It’s tough to convince people about that [the importance of addressing antibiotic resistance]. It’s invisible … And it is real, but it’s invisible. So it’s very difficult to push the message across, people don’t see it you know. They think like, it doesn’t matter, I’m better today, that’s all that matters to me.” (CS16, male, 26 years old)

#### Views on antibiotic resistance

Consumer understanding of antibiotic resistance was conceptualised in four ways: (a) as a property of the body — body becomes resistant to antibiotics; (b) as a property of the medication — antibiotic is no longer effective; (c) as a property of the bacteria — bacteria is resistant to the antibiotic; and (d) as a property of a collective — society is immune to antibiotics. The following quotes illustrate the different ways consumers described antibiotic resistance.

Expressing antibiotic resistance as both a property of the body and of the antibiotic:

“… it’s [the antibiotic] just not effective for the body anymore. That’s obviously, you know, it’s like the body’s built up this immunity to it [the antibiotic] actually working.” (CS03, female, 27 years old)

Expressing antibiotic resistance as a property of the bacteria:

“… bacteria … is getting stronger. … People don’t finish the doses … so … the first part of the treatment there are more weak bacteria [that] died, and then the stronger ones live and as you don’t finish [the course of antibiotics], only the stronger ones [survive] and genetically would be the best bacteria, and that’s what’s happened.” (CS12, male, 25 years old)

Expressing antibiotic resistance as a property of a collective:

“… if it’s [antibiotics] prescribed for reasons which aren’t very serious, then as a society we get immune to the effects of those drugs [antibiotics].” (CS10, female, 23 years old)

Other misconceptions about antibiotics and antibiotic resistance gleaned from this consumer sample were: (a) many were not aware that antibiotic resistance could occur at both an individual and societal level, having assumed it was one or the other, (b) a few were unaware that using antibiotics inappropriately for the common cold and/or coughs can contribute to antibiotic resistance, and in time cause these antibiotics to be ineffective for the treatment of other more serious infections; and (c) most were unaware that resistant bacteria could be transferred from person to person or from food-producing animals to people.

Consumers were concerned as to whether antibiotic resistance could be adequately addressed in a timely manner; pointing out that preventing/reducing antibiotic resistance is preferable to having to find ways to resolve it. A well-informed minority were very concerned about the issue:

“I’m actually quite worried. ‘Coz even healthy people, when you travel around, one of the main reasons for spread of all these drug resistant strains has been human movement. … people can carry them [resistant strains of bacteria] with them, and not- [be sick], just a carrier. And then, you know, you go to a region that’s endemic for … these drug resistant bacteria, pick them up and you come back, and you disseminate it. And that’s how it spread[s]. So, yeah, I’m, I’m quite worried. And I hope if I do get a bacterial infection, it’s not drug resistant.” (CS15, female, 28 years old).

On the other hand, some consumers were optimistic and confident that medical technologies to address or overcome resistant bacteria would soon be found:

“I guess medicine and technology is advancing so quickly that they may be able to stamp them [resistant bacteria] all out …” (CS09, female, 31 years old); and

“Scientists out there will come up with something and they’re really clever, so I don’t worry too much because I think somebody’s solving the problem.” (CS23, female, 28 years old)

Consumers who had lived and/or worked overseas felt that Australia had been managing the issue of antibiotic resistance rather well. They note that antibiotics are regulated prescription medicines in Australia; they surmised and were optimistic that GPs acted as effective gatekeepers for antibiotic use in the community. There was also a sense of complacency, of being safe in a “first-world” country which conferred a false sense of impermeability to issues such as antibiotic resistance.

#### Mitigating antibiotic resistance

Many consumers felt that GPs should play a more proactive role e.g. prescribe less antibiotics, educate patients when antibiotics are not required, and not succumb to patient demands for antibiotics. However, they acknowledged that consumers needed to be part of the solution. At the personal/individual level, consumers felt that by avoiding unnecessary antibiotics they were not worsening the problem of antibiotic resistance. When antibiotics were prescribed, consumers recognised that socially responsible behaviour on their part would constitute completing the course of antibiotics, so as not to encourage the growth of resistant bacteria. At the societal level, consumers reported attempting to influence their social and familial circle, albeit with mixed results.

Consumers maintained a realistic view of having to tackle antibiotic resistance from multiple angles, highlighting the need for conservation of antibiotics now through individual efforts, while at the same time pursuing innovative approaches:

“… So we need to look after what we have, and not just hope that the next breakthrough is just around the corner. I certainly hope that it is. But … where does it stop? You might find a new antibiotic, well that one becomes resistant … so I think it’s a continual thing.” (CS19, female, 31 years old)

## Discussion

This study adds to current literature in the field the perspectives, attitudes and behaviours of Australian consumers toward antibiotic use and antibiotic resistance. In particular, consumer information needs regarding prescribed antibiotics, consumer expectations and perceived trustworthiness of a GP consult, the importance of GP-mediated advice on antibiotic use behaviours, and consumer views and behaviours in relation to delayed antibiotic prescriptions.

Australian consumers expect to be given information regarding prescribed antibiotics which would enable appropriate use and motivate adherence. Consumers want information on how the antibiotic worked; the GP’s rationale for antibiotic selection; treatment duration; the rationale for completing the course of treatment or the consequences of not doing so; and advice regarding alcohol consumption while on antibiotics. Consumers sought other avenues of information when not enough detail was provided as evidenced by a retrospective analysis of an Australian medicines helpline where over 40% of antibiotic-related calls were due to inadequate information provision [35]. Community pharmacists are well-placed to provide medicines information to consumers, and should proactively seek to do so when consumers present an antibiotic prescription for dispensing [36]. However, consumers interviewed in this study reported that while they did not ask, detailed information was not voluntarily provided by either the GP or community pharmacist apart from the product information sometimes included with the antibiotic. An in-depth exploration of consumer perception of the role of community pharmacists in mitigating antibiotic resistance was not part of the scope of this study.

The expectations of Australian consumers of a GP consult are made explicit in this study. Consumers want to be listened to; to be given a thorough clinical examination; to have the GP explain their findings, treatment options, and decisions including the decision not to treat with antibiotics when it is not warranted; and to have their questions answered. These elements of the consultation, when present, increased consumer confidence in the professionalism, competence and trustworthiness of the GP. Most consumers would accept the GP’s decision not to prescribe an antibiotic if it was clearly explained, which complements findings from other studies where patient satisfaction with clinic visits were not necessarily related to getting antibiotics [21, 37]. Many simply wanted reassurance that they did not have a more serious illness requiring treatment, were seeking information, or required pain relief [21]. Consumers who have an established doctor-patient relationship with a GP tend to have shared expectations regarding the overall management of their health, including avoiding antibiotics where possible.

GP-mediated advice exerted an important influence in encouraging desirable antibiotic use behaviours in consumers and preventing inappropriate behaviours, even as consumer behaviours in self-care and antibiotic use were shaped through the social influences of significant others (partner and family), friends, and workplace culture. Hence, GPs need to be empowered and skilled to communicate confidently and unambiguously to patients, especially when conveying the decision that an antibiotic is not warranted and discussing other management options. Public health campaigns can be used to support GPs in their antibiotic stewardship role, by reframing public perception as to what constitutes a “good” GP, contextualised for conservation of antibiotics. In this study, consumer expectations of a GP consult outlined earlier, mirrored the qualities of a “good” GP reported by Australian GPs — someone who: has the skills to deal with uncertainty; practices evidence-based medicine where evidence is available; has good communication skills; has the ability to establish rapport to build robust doctor-patient relationships; and makes treatment decisions in the best interest of the patient [38]. Hence, when antibiotics are not warranted, “… good GPs talk to you about not using antibiotics” [38].

Regarding delayed antibiotic prescriptions, consumers had mixed views on the use of this strategy when there was uncertainty in diagnosis. While some welcomed the convenience of this avenue of accessing antibiotics, others did not want the responsibility of being the final decision maker. Overall, if such prescriptions were issued consumers wanted specific instructions from GPs on when/whether to use the prescription; a finding which corroborates that of a recent Australian study focussed on delayed antibiotic prescribing [39].

Consumer interpretation of the GP’s intention through the provision of delayed antibiotic prescriptions led some to assume that the GP was leaning towards the judgment that antibiotics were warranted. Such interpretation prompted consumers to immediately fill the prescription. This behaviour highlights the differences between consumer and GP interpretation and tolerance of clinical uncertainty; and negates the GP’s intent to take a “watch and wait” approach. Hence, while there is evidence that delayed antibiotic prescriptions could reduce antibiotic consumption [40], policy failure is foreseeable unless mismatched interpretations are resolved through clear communication.

Unused antibiotic prescriptions create a problematic “reservoir” of antibiotics in the community, which could potentially be misused. Consumers reported retaining unused antibiotic prescriptions for future use, discarding them only when the prescription can no longer be dispensed. Hence, regulatory changes to the national medicines subsidy scheme to remove oral antibiotic repeats and to reduce the period of prescription validity should be enacted [41]; while ensuring accessibility of antibiotics where there is a proven clinical need.

Despite the consumer sample being highly educated, there was variation in the level of knowledge, awareness and concerns regarding antibiotic use and antibiotic resistance; some of which were erroneous indicating that consistently clear messages from health professionals, public health campaigns, and media are needed. Australian consumers conceptualised antibiotic resistance in four ways. Three conceptualisations are similar to consumers in Europe [14, 15] and the fourth is new: antibiotic resistance understood as a property of a collective — society is immune to antibiotics. Despite conceptualising antibiotic resistance as a property of a collective, several “blind spots” in antibiotic awareness related to this concept were found — being unaware of the individual and societal consequences of using antibiotics inappropriately for the common cold/cough, and that resistant bacteria could be transferred from person to person. It is sobering to realise that consumers with perhaps less formal education and/or less awareness of the topic may hold more misconceptions about antibiotic use and antibiotic resistance. In beginning to generally address consumer misconceptions, future public health campaigns should adopt clearer terminology — using “antibiotic resistant infections” or “antibiotic resistant bacteria” rather than simply “antibiotic resistance”; and include key messages that highlight the interdependence of individual action and societal consequences. For example, clarify that inappropriate use of antibiotics for minor self-limiting illnesses would in time cause these antibiotics to be ineffective for the treatment of more serious infections; that antibiotic resistance occurs at both an individual and societal level; and that resistant bacteria can be passed to others, potentially harming those with frail health.

### Future research

Given the findings of this study, questions remain on the efficacy and cost-effectiveness of public health campaigns in influencing consumer behaviour; and the extent of behaviour change possible through the re-engineering of social and cultural memes. Research in these areas should be supported.

### Strengths and limitations

Semi-structured interviews captured participants’ knowledge, lived experience and views, in their own words; which helped overcome potential researcher bias and resulted in the co-creation of knowledge [30, 42]. Interviews were conducted one-on-one which allowed participants to engage in unfettered commentary and removed any social pressure to conform to other views being expressed.

The use of convenience and snowball sampling meant that only respondents with interest in the topic volunteered to participate. Other consumers may hold different views. Recruitment was limited to the university population which is not likely to be representative of the general population in terms of educational levels achieved. However, the university is a microcosm of diversity as demonstrated by the consumer sample i.e. multi-ethnic, multi-cultural, and includes people from rural/regional areas, ensuring that a variety of views were captured. This study is relevant to the Australian context; and as is the case with other qualitative studies, generalisability of the findings was not intended.

## Conclusions

Australian consumers expect information on prescribed antibiotics which enable appropriate use, and a GP consult conducted in a manner that increases consumer confidence in the treatment decision. To more fully engage consumers as partners in mitigating antibiotic resistance, consumer information needs regarding prescribed antibiotics must be addressed; shared expectations between consumers and GPs in avoiding the use of antibiotics should be encouraged; resources such as the Return of Unwanted Medicines service should be widely promoted; and the use of clearer terminology and the development of new emphases suggested by this study for public health campaigns should be supported. Regulatory changes to the national medicine subsidy scheme to remove oral antibiotic repeats and to reduce the period of validity for oral antibiotic prescriptions should be enacted. This study provided useful insight into the perspectives, attitudes and behaviours of Australian consumers towards antibiotic use and antibiotic resistance, and presented pertinent suggestions for Australian public health policy and practice.

## Abbreviations

GP: General practitioner
OECD: Organisation for Economic Co-operation and Development
WHO: World Health Organization

## References

[1] Phelps CE (1989). Bug/drug resistance: sometimes less is more. Med Care.

[2] What is antimicrobial resistance? [http://www.who.int/features/qa/75/en/]. Accessed 28 Sept 2017.

[3] OECD: Health at a glance 2015: OECD indicators. 2015.

[4] Department of Health, Health Do (2013). Australian statistics on medicine 2011. In.

[5] Belongia E, Naimi T, Gale C, Besser R (2002). **Antibiotic use and upper respiratory infections: a survey of knowledge, attitudes, and experience in Wisconsin and Minnesota**. Prev Med.

[6] NPS MedicineWise. Two in three Aussie workers incorrectly believe antibiotics work for colds and flu [Media release]; 2014.

[7] Braun B, Fowles J (2000). Characteristics and experiences of parents and adults who want antibiotics for cold symptoms. Arch Fam Med.

[8] Eng J, Marcus R, Hadler J, Imhoff B, Vugia D, Cieslak P, Zell E, Deneen V, McCombs K, Zansky S (2003). Consumer attitudes and use of antibiotics. Emerg Infect Dis.

[9] Gonzales R, Wilson A, Crane L, Barrett PJ (2000). What's in a name? Public knowledge, attitudes, and experiences with antibiotic use for acute bronchitis. Am J Med.

[10] McDonnell Norms Group (2008). Antibiotic overuse: the influence of social norms. J Am Coll Surg.

[11] Godycki-Cwirko M, Nocun M, Butler CC, Muras M, Fleten N, Melbye H (2011). Sickness certification for patients with acute cough/LRTI in primary care in Poland and Norway. Scand J Prim Health Care.

[12] McCullough AR, Parekh S, Rathbone J, Del Mar CB, Hoffman TC (2015). A systematic review of the public's knowledge and beliefs about antibiotic resistance. J Antimicrob Chemother.

[13] Brooks L, Shaw A, Sharp D, Hay A (2008). Towards a better understanding of patients' perspectives of antibiotic resistance and MRSA: a qualitative study. Fam Pract.

[14] Brookes-Howell L, Elwyn G, Hood K, Wood F, Cooper L, Goossens H, Ieven M, Butler CC (2012). The body gets used to them': Patients' interpretations of antibiotic resistance and the implications for containment strategies. J Gen Intern Med.

[15] Wellcome Trust (2015). Exploring the consumer perspective on antimicrobial resistance.

[16] Cals JW, Boumans D, Lardinois RJ, Gonzales R, Hopstaken RM, Butler CC, Dinant GJ (2007). Public beliefs on antibiotics and respiratory tract infections: an internet-based questionnaire study. Br J Gen Pract.

[17] Huttner B, Goossens H, Verheij T, Harbarth S (2010). Characteristics and outcomes of public campaigns aimed at improving the use of antibiotics in outpatients in high-income countries. Lancet Infect Dis.

[18] NPS MedicineWise. Three ways to protect yourself from a 'superbug plague' [Media release]; 2013.

[19] Céspedes A, Larson E (2006). Knowledge, attitudes, and practices regarding antibiotic use among Latinos in the United States: review and recommendations. Am J Infect Control.

[20] World Health Organization (2014). Antimicrobial resistance: Global report on surveillance.

[21] Butler C, Rollnick S, Pill R, Maggs-Rapport F, Stott N (1998). Understanding the culture of prescribing: qualitative study of general practitioners' and patients' perceptions of antibiotic for sore throats. BMJ.

[22] Anghel IB, Craciun C (2013). Self-medication with over-the-counter drugs and antibiotics in Romanian consumers: a qualitative study. Cogn Brain Behav.

[23] Hawkings NJ, Wood F, Butler CC (2007). Public attitudes towards bacterial resistance: a qualitative study. J Antimicrob Chemother.

[24] Norris P, Chamberlain K, Dew K, Gabe J, Hodgetts D, Madden H (2013). Public beliefs about antibiotics, infection and resistance: a qualitative study. Antibiotics.

[25] World Health Organization (2015). Global action plan on antimicrobial resistance.

[26] Australian Government: Responding to the threat of antimicrobial resistance: Australia's first National Antimicrobial Resistance Strategy 2015–2019. Canberra, Australia: Australian Government; 2015.

[27] Australian Government, Department of Health, Department of Agriculture and Water Resources (2016). Implementation plan: Australia's first national antimicrobial resistance strategy 2015–2019.

[28] Biesta G, Tashakkori A, Teddlie C (2010). Pragmatism and the philosophical foundations of mixed methods research. SAGE handbook of mixed methods in social & behavioural research.

[29] Australia's welfare. [https://www.aihw.gov.au/reports/australias-welfare/australias-welfare-2015-inbrief/contents/working-age]. Accessed 6 Oct 2017.

[30] Rubin HJ, Rubin IS (2012). Qualitative interviewing: the art of hearing data.

[31] Miles MB, Huberman AM, Saldana JM (2014). Qualitative data analysis: a methods sourcebook.

[32] Jefferson G, Atkinson J, Heritage J (1984). Transcription notation. Structures of social action: studies in conversation analysis.

[33] NVivo Pro (Version 11.3.1.777) [Computer software]. In*.* Burlington, MA: QSR International; 2016.

[34] Saldana J (2013). The coding manual for qualitative researchers.

[35] Hawke KL, McGuire TM, Ranmuthugala G, van Driel ML (2016). What do consumers want to know about antibiotics? Analysis of a medicines call centre database. Fam Pract.

[36] World Health Organization (2014). The role of pharmacist in encouraging prudent use of antibiotics and averting antimicrobial resistance: a review of policy and experience in Europe.

[37] Coenen S, Francis N, Kelly M, Hood K, Nuttall J, Little P, Verheij TJ, Melbye H, Goossens H, Butler CC (2013). Are patient views about antibiotics related to clinician perceptions, management and outcome? A multi-country study in outpatients with acute cough. PLoS One.

[38] Lum EPM (2017). Making decisions about antibiotic use in the Australian primary healthcare sector (doctoral thesis).

[39] Sargent L, McCullough A, Del Mar C, Lowe J. Using theory to explore facilitators and barriers to delayed prescribing in Australia: a qualitative study using the theoretical domains framework and the behaviour change wheel. BMC Fam Pract. 2017;18(20). https://doi.org/10.1186/s12875-017-0589-1. 10.1186/s12875-017-0589-1 PMC5307801 28193174

[40] Sargent L, McCullough A, Del Mar C, Lowe J (2016). Is Australia ready to implement delayed prescribing in primary care? A review of the evidence. Aust Fam Physician.

[41] Department of Health, Pharmaceutical Benefits Advisory Committee (2015). March 2015 PBAC meeting - consideration of the report of the drug Utlisation sub-committee.

[42] Kvale S (2007). Doing interviews.

